# IL-7 signalling represses Bcl-6 and the T_FH_ gene program

**DOI:** 10.1038/ncomms10285

**Published:** 2016-01-08

**Authors:** Paul W. McDonald, Kaitlin A. Read, Chandra E. Baker, Ashlyn E. Anderson, Michael D. Powell, André Ballesteros-Tato, Kenneth J. Oestreich

**Affiliations:** 1 Virginia Tech Carilion Research Institute, Roanoke, Virginia 24016, USA; 2 Division of Clinical Immunology and Rheumatology, Department of Medicine, University of Alabama at Birmingham, Birmingham, Alabama 35294, USA; 3 Department of Biomedical Sciences and Pathobiology, Virginia-Maryland Regional College of Veterinary Medicine, Virginia Tech, Blacksburg, Virginia 24061, USA; 4 Virginia Tech Carilion School of Medicine, Roanoke, Virginia 24016, USA

## Abstract

The transcriptional repressor Bcl-6 is linked to the development of both CD4^+^ T follicular helper (T_FH_) and central memory T (T_CM_) cells. Here, we demonstrate that in response to decreased IL-2 signalling, T helper 1 (T_H_1) cells upregulate Bcl-6 and co-initiate T_FH_- and T_CM_-like gene programs, including expression of the cytokine receptors IL-6Rα and IL-7R. Exposure of this potentially bi-potent cell population to IL-6 favours the T_FH_ gene program, whereas IL-7 signalling represses T_FH_-associated genes including *Bcl6* and *Cxcr5*, but not the T_CM_-related genes *Klf2* and *Sell*. Mechanistically, IL-7-dependent activation of STAT5 contributes to Bcl-6 repression. Importantly, antigen-specific IL-6Rα^+^IL-7R^+^ CD4^+^ T cells emerge from the effector population at late time points post influenza infection. These data support a novel role for IL-7 in the repression of the T_FH_ gene program and evoke a divergent regulatory mechanism by which post-effector T_H_1 cells may contribute to long-term cell-mediated and humoral immunity.

## Abstract (web summary)

It remains incompletely understood how cytokines shape T_H_1 cell differentiation to central memory T (T_CM_) and follicular T helper (T_FH_) cells. Here the authors show that T_H_1 cells can co-initiate the expression of both T_FH_ and T_CM_ gene programs and that IL-7 signalling represses T_FH_-associated but not T_CM_-associated genes.

## Graphical abstract

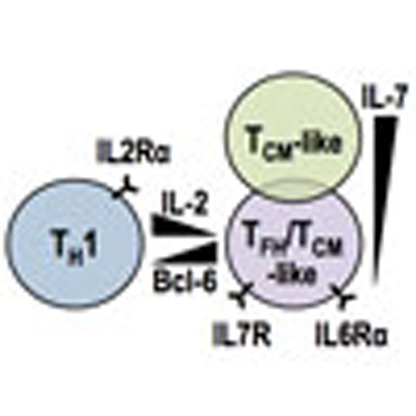

During the course of an immune response, CD4^+^ T helper cells identify invading pathogens, proliferate and secrete cytokines to aid in immune-mediated clearance of infection. This results in an initial expansion of effector CD4^+^ T cells. As pathogen is eliminated, the number of CD4^+^ T cells is reduced to avoid potential autoimmunity that may result from a prolonged effector T-cell response. During this contraction phase, long-lived CD4^+^ T cells—termed memory T cells—survive to respond more quickly and robustly should the immune system re-encounter the same pathogen. The development of comprehensive immunological memory requires the generation of cells that are capable of contributing to both long-term cell-mediated and humoral immune responses, and includes populations of both CD4^+^ central memory T (T_CM_) and T-follicular helper (T_FH_) cells.

T_CM_ cells are one of many CD4^+^ memory T-cell populations that also include effector memory (T_EM_), resident memory (T_RM_) and recirculating memory (T_RCM_) cell types^1^,^2^,^3^. These diverse sets of memory cells have been primarily identified by their immune function, tissue location and cell surface receptor expression. Specifically, T_CM_ cells are uniquely identified by the expression of the homing receptors L-selectin (CD62L, encoded by the gene *Sell*) and Ccr7 (ref. 1). The expression of these two receptors allows for trafficking to the T-cell zones of secondary lymphoid areas where T_CM_ cells can participate in cell-mediated immunity by engaging in antigen surveillance.

T_FH_ cells facilitate humoral immune responses by assisting B-lymphocytes with the production of pathogen-neutralizing antibodies^4^,^5^,^6^. T_FH_ cells are defined in part by the expression of the cell surface receptor Cxcr5, which allows for homing to the B cell zones of secondary lymphoid organs^4^,^5^. Differentiation of the T_FH_ cell subset is a complex and multi-step process. These steps include the initiation of the T_FH_ gene program, denoted by an initial upregulation of a partial T_FH_-profile (that is, Bcl-6 and Cxcr5 expression), followed by a second stage, whereby full commitment to the germinal center T_FH_ cell state occurs upon B-cell interaction and enhanced ICOS signalling^7^. Given the inherent complexity of this process, many questions remain regarding the genesis of T_FH_ cell populations. For example, while T_FH_ cells can and do develop during the initial response to antigen, it has also been demonstrated that other effector T-helper cell subsets are capable of adopting a T_FH_-like profile, making a ‘post-effector' developmental pathway plausible as well^8^,^9^,^10^,^11^,^12^,^13^. These previous studies are important as they support the possibility that, in addition to effector T_FH_ cells, other T-helper populations may assist in long-term antibody-mediated immunity by co-opting certain aspects of the T_FH_-cell gene program.

It has been previously demonstrated that the transcriptional repressor Bcl-6 is required for T_FH_ development^14^,^15^,^16^. Bcl-6 directs T_FH_ differentiation, at least in part, by antagonizing the expression of a second transcriptional repressor, Blimp-1, a known negative regulator of T_FH_ cell differentiation^12^,^14^,^15^,^16^. Interestingly, upregulation of Bcl-6 has also been implicated in the differentiation of CD4^+^ effector T cells into memory cells, including the T_CM_ subset^17^,^18^,^19^,^20^. However, it is currently unclear how this increase in Bcl-6 expression initiates this effector-to-memory transition. As with T_FH_ differentiation, it has been postulated that a key function of Bcl-6 in promoting memory cell formation is to repress the expression of Blimp-1, a factor positively linked to the terminal differentiation of the effector cell state^2^,^21^,^22^,^23^. However, whether there are additional roles or long-term requirements for Bcl-6 expression in establishing T_CM_-cell fate apart from Blimp-1 repression remains unclear.

Although transcription factors such as Bcl-6 are responsible for regulating the gene expression profiles of developing cells, upstream environmental signals, often in the form of a cytokine-dependent response, regulate the expression and/or the functional activity of individual transcription factors^24^. For example, it has been demonstrated that strong interleukin-2 (IL-2) signalling inhibits T_FH_ formation by controlling the expression of Bcl-6 and Blimp-1 (refs 12, 25, 26, 27). Similarly, it has been shown that inflammatory cytokine environments, including those that are IL-2 rich, can inhibit memory cell formation^28^,^29^.

Collectively, evidence in the literature suggests that, despite their distinct functions and localization during the adaptive immune response, T_FH_ and T_CM_ cells share specific regulatory requirements and developmental programs. In addition, several recent studies have reported a high degree of similarity between T_FH_ and T_CM_ cells at the transcriptional and protein expression levels^9^,^17^,^19^,^30^. Furthermore, populations of memory T_FH_ and T_CM_ cells similarly express a number of cell surface receptors including Cxcr5, CD62L and Ccr7 (refs 8, 9, 30, 31, 32). Thus, there are precedents supporting the possibility that these two immune cell populations may be developmentally linked. However, as the contribution of each of these cells to long-term immunity is functionally distinct, it is likely that at some point there is a divergence to unique cytokine and transcriptional networks to allow for specialized T_FH_- and T_CM_-dependent immune responses.

Here, we demonstrate that T-helper 1 (T_H_1) cells are capable of co-initiating the expression of both T_FH_ and T_CM_ gene programs in response to decreased IL-2 signalling. Mechanistically, the initial co-expression of the T_FH_ and T_CM_ programs is due to the Bcl-6-dependent repression of Blimp-1. In addition, during this post-effector stage, T_H_1 cells downregulate IL-2Rα expression, while upregulating the dual expression of IL-6Rα and IL-7R. This apparent cytokine receptor reprogramming results in a population of bi-potent ‘T_FH_/T_CM_-like' cells with the ability to respond to either IL-6 or IL-7. Strikingly, while treatment with IL-6 results in the upregulation of Bcl-6 and Cxcr5 expression, exposure of these cells to IL-7 results in a dose-dependent decrease in T_FH_-associated genes including Bcl-6, while allowing the continued expression of T_CM_-specific genes. Importantly, at late time points post influenza infection, a population of antigen-specific IL-6Rα^+^IL-7R^+^ CD4^+^ T cells emerges coincident with a decline in the effector population. Thus, this study describes a novel role for IL-7 in the repression of the T_FH_ gene program and defines a potential mechanism by which post-effector T_H_1 cells may be able to support aspects of both long-term cell-mediated and humoral immunity.

## Results

### IL-2 signalling modulates T_H_1, T_FH_ and T_CM_ gene expression

**Figure 1:**
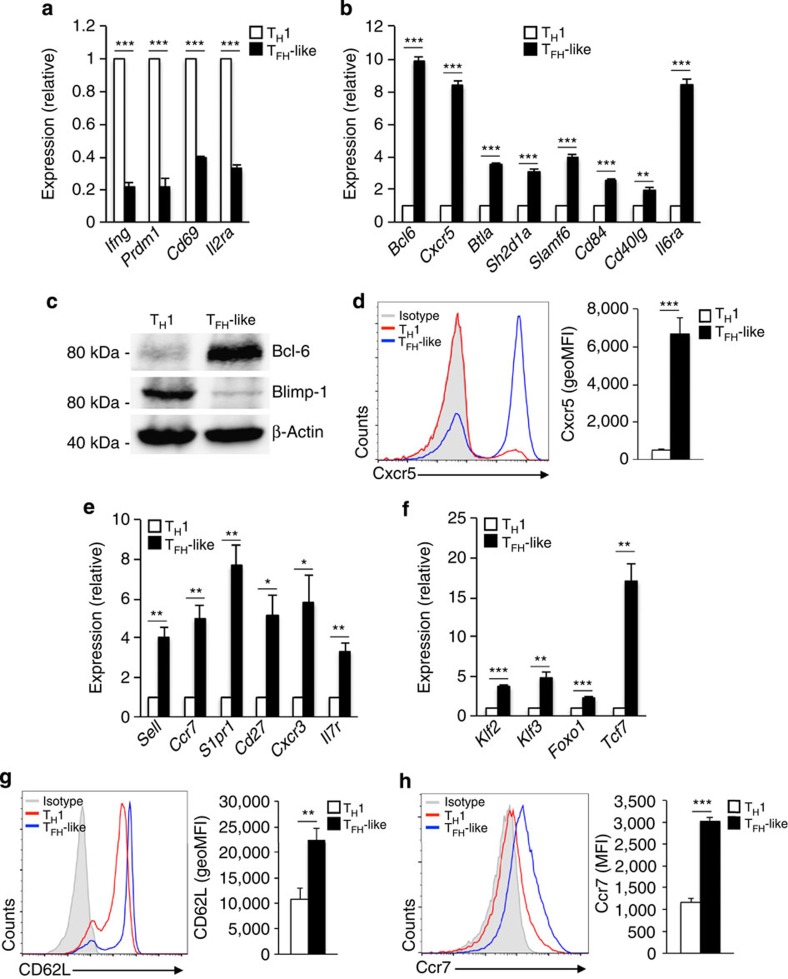
IL-2 signalling regulates Bcl-6 expression in post-effector T_H_1 cells to allow for the upregulation of T_FH_- and T_CM_-like profiles. Primary CD4^+^ T cells were cultured in T_H_1 conditions and exposed to either high (T_H_1 cells) or low (T_FH_-like cells) environmental IL-2 (250 U ml^−1^ or 10 U ml^−1^, respectively). (**a**,**b**) RNA was isolated from the T_H_1 (white bar) and T_FH_-like (black bar) cells and the expression of the indicated genes was determined by quantitative RT–PCR. Data were normalized to *Rps18* as a control and the results are represented as fold change in expression relative to the T_H_1 sample (mean of *n*=3±s.e.m.). (**c**) An immunoblot analysis was performed to assess changes in protein expression in response to alterations of environmental IL-2. Expression for Bcl-6 and Blimp-1 was measured with β-actin serving as a control for equal protein loading. Shown is a representative blot of three independent experiments performed. (**d**) Representative histogram overlay of cell surface expression of Cxcr5 for T_H_1 and T_FH_-like cells. Geometric mean fluorescence intensity (geoMFI) for Cxcr5 is also shown (mean of *n*=5±s.e.m.). (**e**,**f**) qRT–PCR analysis examining expression of the indicated genes in T_H_1 and T_FH_-like cells. Data were normalized and represented as in **a** and **b** (mean of *n*=3±s.e.m.). (**g**,**h**) Representative histogram overlay of cell surface expression of (**g**) CD62L and (**h**) Ccr7 for T_H_1 and T_FH_-like cells. Average geoMFI or MFI for CD62L and Ccr7 expression are shown (mean of *n*=5 or 4±s.e.m.). *P<0.05, **P<0.01, ***P<0.001 (unpaired Student's *t*-test).

Our previous studies demonstrated that reduced IL-2 signalling has the potential to regulate T-helper cell fate decisions by augmenting the expression of the T_FH_ lineage-defining factor Bcl-6 (ref. 12). In addition to its required role in T_FH_ cell development, Bcl-6 expression has also been implicated in the formation of memory CD4^+^ T cells^1^,^17^,^19^,^20^. As such, we examined whether the IL-2-sensitive increase in Bcl-6 expression would similarly result in the upregulation of a memory cell gene program by exposing *in vitro* generated T_H_1 cells to both high and low IL-2 concentrations. As with our prior study, the expression of a number of T_H_1 genes was decreased in low environmental IL-2 conditions, while the expression of key T_FH_ genes was induced, including the expression of *Bcl6* and *Cxcr5* (Fig. 1a,b). Consistent with the increase in transcript, Bcl-6 protein and cell surface expression of Cxcr5 were significantly increased (Fig. 1c,d). Interestingly, in addition to the induction of the T_FH_-like profile, T_H_1 cells exposed to a low IL-2 concentration also upregulated genes associated with the T_CM_ cell type—most notably the lymph node homing receptors *Sell* and *Ccr7* (Fig. 1e)^1^,^2^,^33^. We also observed increased expression of other memory T-cell-related markers (*Il7r, S1pr1*, *Cd27* and *Cxcr3*) and transcription factors known to promote the memory T-cell fate (*Klf2*, *Klf3 and Foxo1*; Fig. 1e,f). Interestingly, the expression of *Tcf7*, the gene that encodes the transcriptional regulator TCF-1, was significantly increased (Fig. 1f). Similar to Bcl-6, the expression of TCF-1 has been implicated in the development of both T_FH_ and memory cells^34^,^35^,^36^,^37^,^38^. Importantly, the induction of the T_CM_-like profile was not limited to changes in transcript alone, as the cell surface expression of both CD62L and Ccr7 was significantly increased compared with that observed in effector T_H_1 cells (Fig. 1g,h). Collectively, these data demonstrate that increased Bcl-6 expression in T_H_1 cells, in response to decreased IL-2 signalling, results in the induction of not only a T_FH_-like profile, but a T_CM_-like profile as well. Furthermore, these data suggest that a common Bcl-6-dependent regulatory mechanism may be responsible for the initiation of both the T_FH_ and T_CM_ gene programs.

### Blimp-1 inhibits the T_CM_-associated genes *Sell* and *Ccr7*

**Figure 2:**
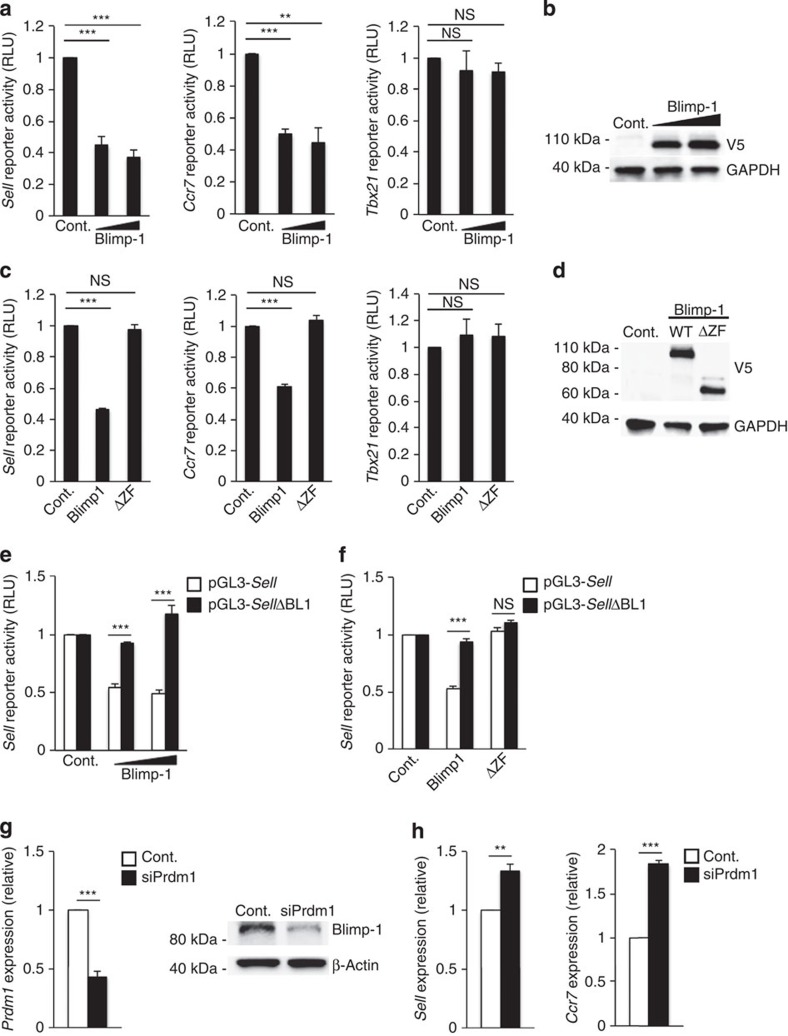
Blimp-1 represses the CD4^+^ T_CM_ genes *Sell* and *Ccr7*. (**a**,**c**,**e**,**f**) EL4 T cells were transfected with the indicated promoter–reporter constructs in combination with a wild-type Blimp-1 expression vector, a Blimp-1 mutant incapable of binding to DNA (ΔZF), or an empty vector control (mean of *n*=3±s.e.m.). In **e** and **f**, EL4 T cells were transfected with either a wild-type *Sell* promoter–reporter or a *Sell* promoter–reporter lacking the predicted Blimp-1 DNA-binding elements (pGL3-*Sell*ΔBL1). Luciferase promoter–reporter values were normalized to a *renilla* control and expressed relative to the control sample for each experiment (mean of *n*=4±s.e.m.). (**b**,**d**) Wild-type and mutant Blimp-1 protein levels were measured by immunoblot analysis. Shown is a representative blot of three independent experiments performed. (**g**,**h**) T_H_1 cells were nucleofected with either siRNA specific to Blimp-1 (siPrdm1) or a control siRNA. Following a 24-h time period, RNA was harvested and expression of *Sell* or *Ccr7* was assessed by qRT–PCR. The data are presented as fold change in expression relative to the control sample (mean of *n*=4±s.e.m.). **P<0.01, ***P<0.001 (unpaired Student's *t*-test). Cont., control; NS, not significant; WT, wild type.

We previously identified Blimp-1 as a factor responsible for directly repressing members of the T_FH_ gene program including *Cxcr5* and *Il6ra*^12^. A key finding from our prior studies was that in response to reduced IL-2 signalling, Bcl-6 expression increased in T_H_1 cells, resulting in the Bcl-6-dependent repression of Blimp-1 and the subsequent induction of the T_FH_ gene program (Fig. 1c)^12^,^39^. Given our finding that increased Bcl-6 expression also leads to the induction of a T_CM_-like profile, we hypothesized that the same mechanistic interplay between Bcl-6 and Blimp-1 that results in the promotion of the T_FH_ program may also initiate the transition from an effector T_H_1 to a T_CM_-like cell state. Therefore, we examined whether T_CM_ genes, similar to T_FH_ genes, were direct Blimp-1 targets in effector T_H_1 cells. Using predictive transcription factor binding site software (Genomatix), we identified potentially functional Blimp-1 DNA-binding elements in the promoters of both *Sell* and *Ccr7*. To determine whether the predicted elements were functional, we prepared promoter–reporter constructs encompassing the predicted sites and performed luciferase reporter experiments in the presence and absence of overexpressed Blimp-1. Importantly, the expression of Blimp-1 resulted in a decrease in the promoter activity of both *Sell* and *Ccr7* (Fig. 2a,b). As a control, there was no Blimp-1-mediated repression of a *Tbx21*-reporter, which lacks predicted Blimp-1 DNA-binding elements. To determine whether Blimp-1-mediated repression may be the result of direct DNA binding, we performed promoter–reporter experiments with a mutant Blimp-1 construct lacking the zinc finger DNA-binding domain (ΔZF). Although wild-type Blimp-1 readily repressed promoter activity, there was no repression with the Blimp-1ΔZF protein (Fig. 2c,d). As a further test of the functional nature of the predicted sites, a *Sell*-reporter construct lacking the Blimp-1 DNA-binding element (pGL3-*Sell*ΔBL1) was not repressed by Blimp-1 (Fig. 2e,f).

To examine the extent of Blimp-1-specific repression in an endogenous setting, we utilized a knockdown approach to assess the effect of decreasing Blimp-1 levels on the expression of these genes in effector T_H_1 cells (Fig. 2g). Upon small interfering RNA (siRNA) knockdown of Blimp-1 expression, we observed a modest but significant increase in both *Sell* and *Ccr7* expression (Fig. 2h). Collectively, these data suggest that the T_CM_-associated genes *Sell* and *Ccr7* are repressed by Blimp-1 in effector T_H_1 cells. Furthermore, these findings suggest that the repression of Blimp-1 by Bcl-6 is a critical event for the initiation of both T_FH_ and T_CM_ gene programs in T_H_1 cells.

### T_H_1 cells undergo cytokine receptor reprogramming

T_CM_ and T_FH_ cell differentiation represent complex- and multistep processes that are directed by a litany of factors. A key determinant that influences immune cell differentiation is the cytokine environment to which the cell is exposed, as well as the ability of that cell to sense and respond to its environment through cytokine receptor expression. Our data indicate that effector T_H_1 cells upregulate both T_CM_- and T_FH_-like gene expression patterns in response to a low IL-2 environment. Importantly, IL-2-signalling is known to influence the expression of cytokine receptors^40^. Initially, *Il2ra* is expressed at high levels in the effector T_H_1 cells. However, as these cells transition to a low IL-2 environment, our data demonstrate that *Il2ra* expression decreases, whereas the expression of *Il6ra* and *Il7r* increases (Fig. 1a,b,e). Hence, the predominant cytokine receptor expression pattern changes from one supportive of effector T_H_1 cells, which are responsive to elevated IL-2, to one enriched with IL-6Rα and IL-7R—receptors that respond to cytokines favouring T_FH_ and T_CM_ development, respectively^41^,^42^,^43^,^44^,^45^,^46^. Furthermore, these data are suggestive of the intriguing possibility that three divergent cell types may emerge from the effector T_H_1 population: a pre-T_FH_-like population (IL-6Rα^+^IL-7R^−^), a pre-T_CM_-like population (IL-6Rα^−^IL-7R^+^) and/or a bi-potent pre-T_FH_/T_CM_ (IL-6Rα^+^IL-7R^+^) population that may be capable of transitioning into either cell type.

**Figure 3:**
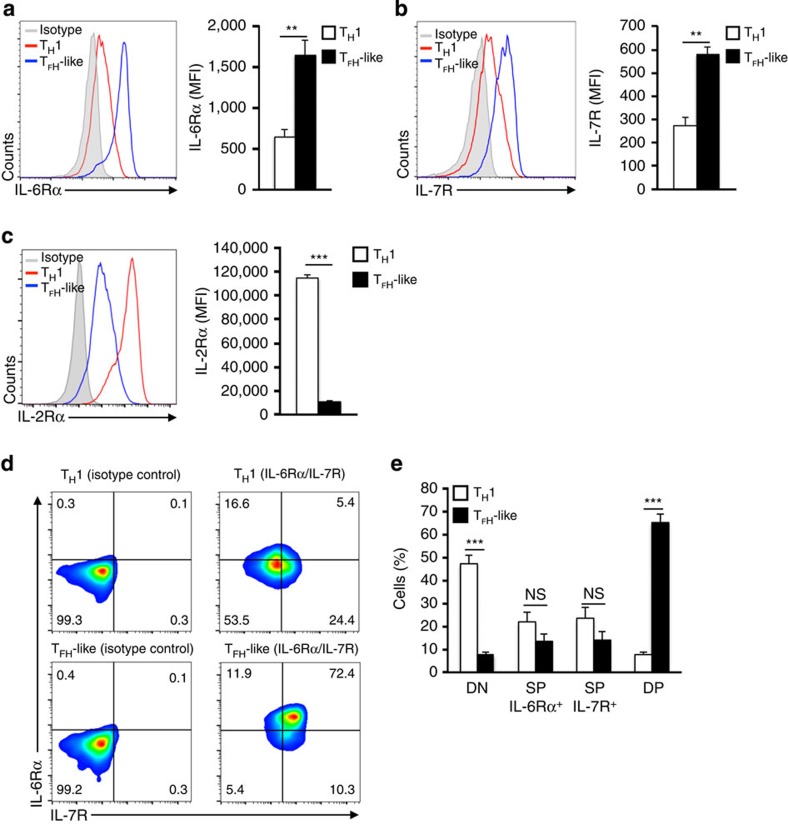
T_H_1 cells undergo cytokine receptor reprogramming to dually express IL-6Rα and IL-7R. Primary CD4^+^ T cells were cultured in T_H_1 conditions and exposed to either high (T_H_1 cells) or low (T_FH_-like cells) environmental IL-2 (250 U ml^−1^ or 10 U ml^−1^, respectively). (**a**–**e**) Cell surface expression of IL-6Rα, IL-7R or IL-2Rα was measured by flow cytometric analysis. Data are represented as histograms (**a**–**c**), flow cytometry dot plots (**d**) or quantified percent positive cells (**e**). Average mean fluorescence intensity (MFI) for IL-6Rα, IL-7R and IL-2Rα expression is shown (mean of *n*=3±s.e.m.). **P<0.01, ***P<0.001 (unpaired Student's *t*-test). DN, double negative; NS, not significant; SP, single positive.

To address the above possibilities, we assessed the composition of both the high IL-2 and low IL-2- treated T_H_1 populations by examining the cell surface expression of IL-6Rα and IL-7R. Consistent with our transcript analysis, the expression of both IL-6Rα and IL-7R was significantly upregulated, whereas IL-2Rα was downregulated, on the surface of the low IL-2-treated cells (Fig. 3a–c). Importantly, the majority of the low IL-2-treated cells displayed dual expression of these receptors (double positive ‘DP', IL-6Rα^+^IL-7R^+^), whereas comparably few of the DP cells were observed in the high IL-2-treated (T_H_1) population (Fig. 3d,e).

**Figure 4:**
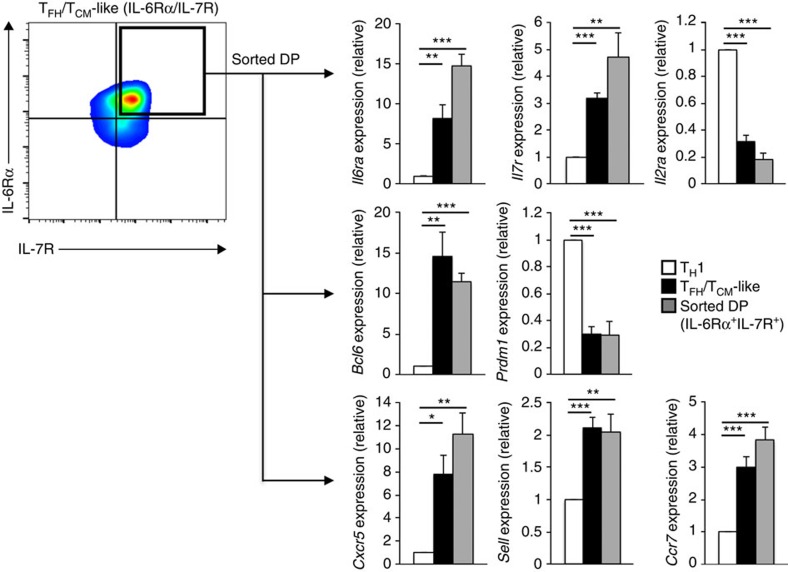
Sorted IL-6Rα^+^IL-7R^+^ double-positive (DP) cells dually express T_FH_- and T_CM_-like gene programs. Droplet digital PCR analysis was used to examine expression of T_H_1-, T_FH_- and T_CM_-associated genes. IL-6Rα^+^IL-7R^+^ double-positive cells were sorted and their expression profiles were compared with that of T_H_1 (High IL-2) and T_FH_/T_CM_-like (Low IL-2) cells. Data were normalized to *Rps18* and are represented as fold change in expression relative to the T_H_1 sample (mean of *n*=4±s.e.m.). *P<0.05, **P<0.01, ***P<0.001 (unpaired Student's *t*-test).

To confirm that the IL-6Rα^+^IL-7R^+^ cells expressed both T_FH_-like and T_CM_-like programs, we sorted the DP population and compared the expression of key T_H_1, T_FH_ and T_CM_ genes to that observed in effector high IL-2-treated (T_H_1) and bulk low IL-2-treated (T_FH_-like) cells. Indeed, while significant differences were observed between the IL-6Rα^+^IL-7R^+^ DP and effector T_H_1 cells, the gene expression programs between sorted DP and bulk T_FH_-like cells were relatively indistinguishable (Fig. 4). Consistent with the transcript analysis, IL-6Rα^+^IL-7R^+^ cells also displayed elevated cell surface expression of CD62L, Ccr7 and Cxcr5 (Supplementary Fig. 1a–c). Collectively, these data support a model whereby, in response to reduced IL-2 signalling, T_H_1 cells co-initiate the expression of both T_FH_ and T_CM_-like gene programs, including the dual expression of IL-6Rα and IL-7R (hereafter referred to as ‘T_FH_/T_CM_-like' cells).

### IL-6 and IL-7 differentially regulate T_FH_ and T_CM_ genes

**Figure 5:**
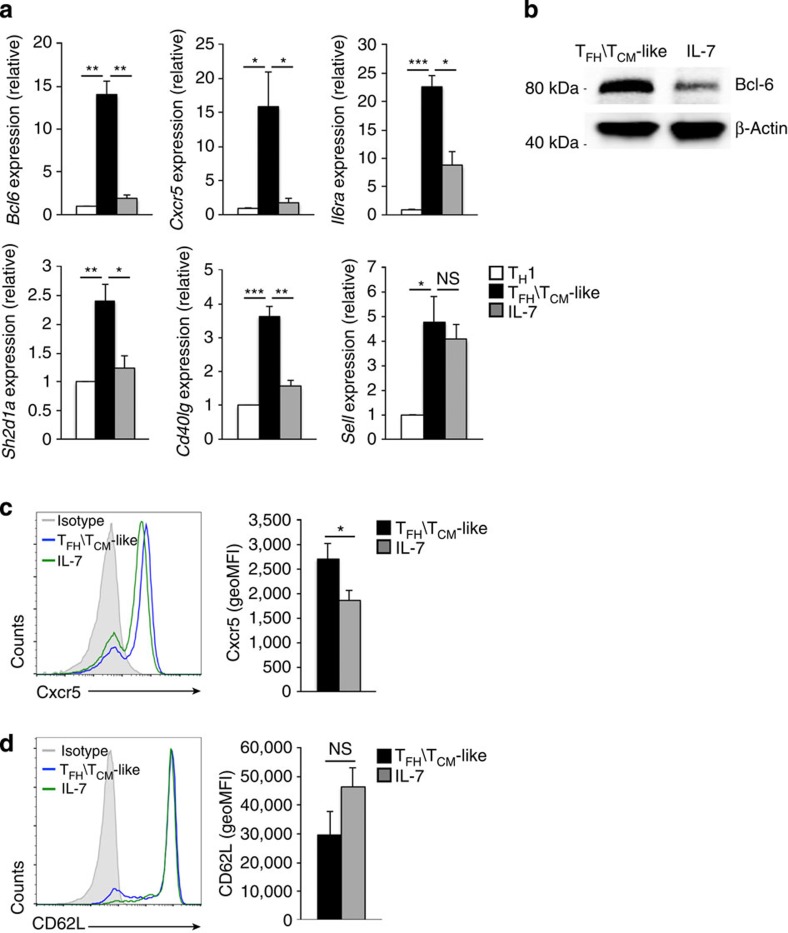
IL-7 signalling represses Bcl-6 and T_FH_ gene expression patterns. Primary CD4^+^ T cells were cultured in T_H_1 conditions and exposed to either high or low environmental IL-2 to generate effector T_H_1 or T_FH_/T_CM_-like (IL-6Rα^+^IL-7R^+^) cells, respectively. T_FH_/T_CM_-like cells were then exposed to IL-7. Following a 24 or 48 h incubation, expression of the indicated genes was measured by (**a**) qRT–PCR, (**b**) immunoblot or (**c**,**d**) flow cytometric analysis. For **a**, the sample values are presented as fold change in expression relative to the T_H_1 sample for each independent experiment (mean of *n*=3±s.e.m.). For **b**, Bcl-6 protein was measured with β-actin serving as a loading control. Shown is a representative blot of three independent experiments performed. For **c** and **d**, data are represented either as histogram flow cytometry plots or quantified geometric mean fluorescence intensity (geoMFI; mean of *n*=5±s.e.m.). *P<0.05, **P<0.01, ***P<0.001 (unpaired Student's *t*-test). NS, not significant.

The cytokines IL-6 and IL-7 have demonstrated roles in establishing and sustaining the T_FH_ and T_CM_ cell fates, respectively^41^,^42^,^43^,^44^,^45^,^46^. Therefore, to test the functional nature of IL-6Rα and IL-7R co-expression, we treated the T_FH_/T_CM_-like cells with either IL-6 or IL-7 and examined the expression of key T_FH_- and T_CM_-associated genes as compared with untreated controls. IL-6 treatment resulted in increased expression of the hallmark T_FH_ genes *Bcl6* and *Cxcr5*, but did not significantly impact the expression of the T_CM_ gene *Sell* (Supplementary Fig. 2a,b). Conversely, exposure to IL-7 resulted in a significant reduction in Bcl-6 expression and the repression of several other T_FH_ genes (*Cxcr5*, *Il6ra*, *Sh2d1a* and *Cd40lg*) (Fig. 5a,b). In many cases, the repression of the T_FH_-associated genes resulted in expression levels near to those observed in effector T_H_1 cells.

As IL-7 is present at low levels in secondary lymphoid tissues, we next exposed the T_FH_/T_CM_-like cells to a range of physiologically relevant IL-7 concentrations and analysed the expression of key T_CM_ and T_FH_ genes (Supplementary Fig. 3)^46^. Strikingly, even at an extremely low concentration of environmental IL-7, hallmark T_FH_ genes (*Bcl6*, *Cxcr5* and *Il6ra*) were preferentially repressed. In stark contrast, the expression of the T_CM_-associated genes *Sell*, *Klf2* and *Ccr7* was relatively unaffected by IL-7 treatment. Importantly, the expression of the anti-apoptotic gene, *Bcl2*, and the gene encoding the glycerol channel aquaporin 9, *Aqp9*, both of which are known to promote the long-term survival of memory cells, was induced in response to IL-7 (Supplementary Fig. 3)^47^,^48^.

To further examine IL-7-induced changes to T_FH_ and T_CM_ cell surface marker expression, we compared Cxcr5 and CD62L protein expression in untreated T_FH_/T_CM_-like cells to those exposed to IL-7. Consistent with our transcript data, IL-7 treatment resulted in a significant reduction in Cxcr5 cell surface expression (Fig. 5c). In contrast, exposure to IL-7 resulted in a modest increase in CD62L, again suggesting that the repressive effect of IL-7 is limited to the T_FH_ profile (Fig. 5d). It is important to note that in spite of the observed decrease in Cxcr5 expression, a majority of the Cxcr5 expressed on the cell surface remained refractory to the IL-7 repressive effect. This was in contrast to the almost complete loss of Bcl-6 protein expression in response to IL-7 treatment. These data corroborate other studies demonstrating that Cxcr5 expression can occur independent of Bcl-6 expression^13^,^49^. Furthermore, these data are consistent with the moderate expression of Cxcr5 observed on T_CM_ cells^1^,^19^,^50^.

### IL-7-induced repression of Bcl-6 is independent of Blimp-1

**Figure 6:**
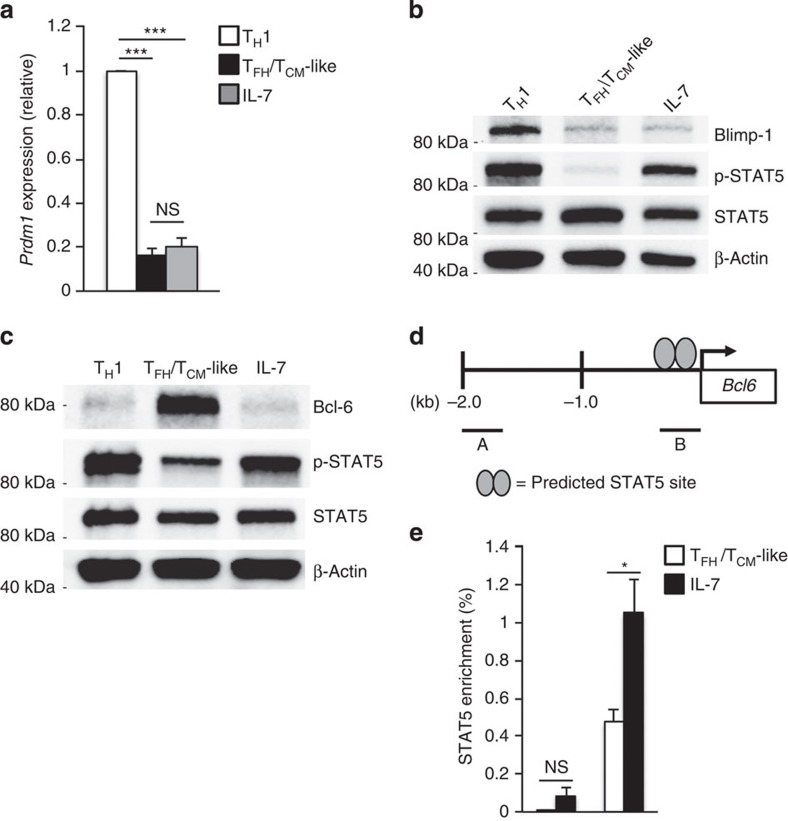
IL-7-induced repression of Bcl-6 is mediated via STAT5 and independent of Blimp-1. (**a**) qRT–PCR or (**b**) immunoblot analysis of Blimp-1 expression in T_H_1, T_FH_/T_CM_-like cells, and T_FH_/T_CM_-like cells exposed to IL-7. In **a**, data were normalized to *Rps18* as a control and the results are presented relative to the T_H_1 sample (mean of *n*=4±s.e.m.). In **b**, protein expression was assessed using the indicated antibody. β-Actin was monitored as a control for equal protein loading. Shown is a representative blot of three independent experiments. (**c**) Immunoblot analysis of Bcl-6, phospho-STAT5 (p-STAT5) and STAT5 expression in primary T_H_1 cells, T_FH_/T_CM_-like cells and T_FH_/T_CM_-like cells exposed to IL-7. β-Actin was monitored to ensure equal protein loading. Shown is a representative blot of three independent experiments. (**d**) A schematic indicating the location of a predicted tetrameric STAT5 binding site in the promoter of the *Bcl6* locus. PCR amplicons used in the chromatin immunoprecipitation (ChIP) analysis are indicated as ‘A' and ‘B'. (**e**) ChIP assay to assess STAT5 association with the *Bcl6* locus in response to IL-7-signalling. Data are represented as percent enrichment relative to a ‘total' input sample (mean of *n*=3±s.e.m.). *P<0.05, ***P<0.001 (unpaired Student's *t*-test). NS, not significant.

The finding that IL-7 represses Bcl-6 expression has potentially far-reaching implications given the demonstrated roles for Bcl-6 in T_FH_ development, T_CM_ differentiation and in the regulation of cellular functions including metabolism and cell cycle progression^14^,^15^,^16^,^20^,^39^,^51^,^52^. As such, we sought to determine the identity of the transcription factor(s) responsible for directing IL-7-mediated repression of Bcl-6. There is a well-established inverse relationship between the transcriptional repressors Bcl-6 and Blimp-1 (refs 12, 14, 17, 53). As our data demonstrate that IL-7-signalling inhibits Bcl-6 expression, we considered the possibility that IL-7 treatment may lead to increased Blimp-1 expression. To test this possibility, we examined the level of *Prdm1* (Blimp-1) expression in T_H_1 cells compared with that of T_FH_/T_CM_-like cells, and T_FH_/T_CM_-like cells exposed to IL-7 (Fig. 6a). Consistent with our previous results, T_H_1 cells displayed significantly elevated expression of *Prdm1* transcript as compared with T_FH_/T_CM_-like cells^12^. Interestingly, despite increased activation of STAT5 (which has been positively linked to Blimp-1 expression in response to IL-2 signalling), no appreciable increase in Blimp-1 protein was observed in the T_FH_/T_CM_-like cells stimulated with IL-7 (Fig. 6b). These data indicate that Blimp-1 is unlikely to be the IL-7-responsive factor that represses Bcl-6 expression. Furthermore, these data suggest that the IL-7-treated T_FH_/T_CM_-like cells are not simply reverting to a short-lived effector T_H_1 phenotype (that is, possessing high Blimp-1 expression). Rather, it appears they are transitioning into cells with a gene program that more closely resembles that of long-lived T_CM_ cells.

### IL-7 regulates STAT5 association with the *Bcl6* promoter

It is well established that the transcription factor STAT5 is activated downstream of IL-7 signalling^46^,^54^,^55^. Although STAT5 is known to function as a transcriptional activator, recent studies have described novel roles for STAT5 in the direct repression of gene expression^12^,^56^. To determine whether IL-7-signalling represses Bcl-6 expression through the activation of STAT5, we compared the activation state of STAT5 (p-STAT5) in effector T_H_1, T_FH_/T_CM_-like cells and T_FH_/T_CM_-like cells exposed to IL-7 (Fig. 6c). Cells treated with IL-7 displayed elevated levels of STAT5 phosphorylation similar to that observed in effector T_H_1 cells. In contrast, T_FH_/T_CM_-like cells had relatively low STAT5 activation. Importantly, the increased level of STAT5 activation in IL-7-treated cells correlated with decreased Bcl-6 expression.

Given the inverse relationship between activated STAT5 and Bcl-6 expression, we hypothesized that STAT5 could play a role in the IL-7-mediated repression of Bcl-6 by directly binding to and repressing the *Bcl6* promoter. In support of this hypothesis, a recent report demonstrated that a DNA sequence-specific tetrameric STAT5 complex functions downstream of IL-7R-signalling to repress gene expression during B-cell differentiation^56^. We analysed the *Bcl6* locus and identified a potential tetrameric STAT5 DNA-binding site in the promoter region (Fig. 6d). We then performed chromatin immunoprecipitation (ChIP) analyses to determine whether STAT5 associates with the *Bcl6* promoter in response to IL-7. Indeed, we detected increased STAT5 binding at the *Bcl6* locus in T_FH_/T_CM_-like cells exposed to IL-7 as compared with untreated T_FH_/T_CM_-like cells (Fig. 6e). Importantly, the highest levels of STAT5 association corresponded to the location of the predicted tetrameric STAT5 binding site (Fig. 6d,e). Collectively, these data support a role for IL-7-activated STAT5 in the direct repression of Bcl-6 expression.

### IL-6Rα^+^IL-7R^+^ CD4^+^ T cells emerge post influenza infection

**Figure 7:**
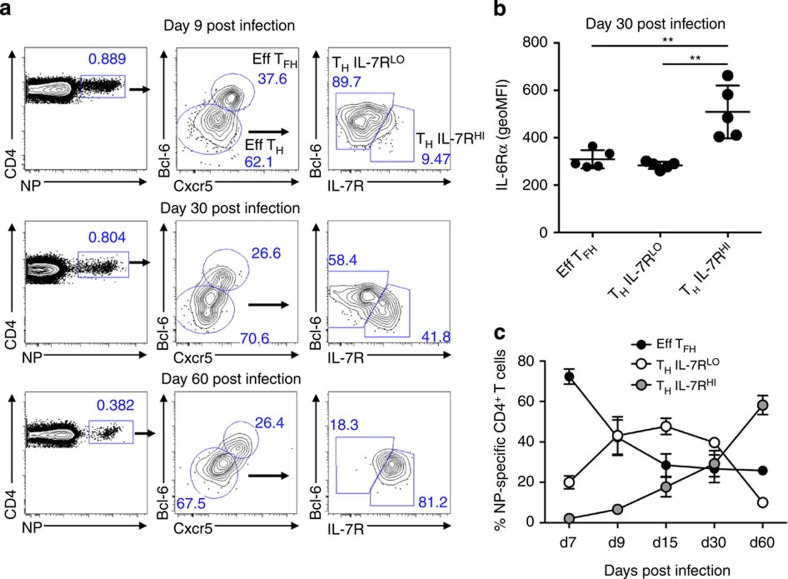
IL-6Rα^+^IL-7R^+^ CD4^+^ T cells are detected at late time points post influenza infection. Mice were infected with influenza (PR8) and CD4^+^CD19^−^Foxp3^−^ cells were analysed by flow cytometric analysis at the indicated day post infection. (**a**) Nucleoprotein (NP)-specific cells were isolated and sorted into effector T_FH_ (Eff T_FH_) and non-T_FH_ (Eff T_H_) populations. Non-T_FH_ effector cells were then assessed for IL-7R expression and further classified as IL-7R low (IL-7R^LO^) or IL-7R high (IL-7R^HI^). (**b**) IL-6Rα expression of the indicated cell population was measured by flow cytometric analysis (geometric mean fluorescence intensity (geoMFI); mean of *n*=5±s.d.). (**c**) Percent NP-specific distribution of Eff T_FH_, T_H_ IL-7R^LO^ and T_H_ IL-7R^HI^ cells at the indicated time points (Day 7, d9, d15, d30, d60) post influenza infection (mean of *n*=5±s.d.). **P<0.01 (unpaired Student's *t*-test).

A key discovery from our *in vitro* experiments was that effector T_H_1 cells upregulate both T_FH_ and T_CM_ gene programs in response to withdrawal of IL-2. These conditions are consistent with the late stages of an immune response, as the pro-inflammatory cytokine environment wanes and the effector population transitions to a memory population capable of supporting both cell-mediated and humoral immunity. Intriguingly, we observed that effector T_H_1 cells undergo a period of cytokine receptor reprogramming in which they downregulate IL-2Rα, upregulate IL-6Rα and IL-7R, and initiate the expression of hallmark T_FH_ and T_CM_ genes. Therefore, to assess the kinetics of IL-6Rα and IL-7R expression *in vivo*, we infected mice with influenza (A/PR8/34; ‘PR8') and monitored antigen-specific (nucleoprotein, ‘NP'-specific) CD4^+^ T cells at multiple time points post infection (days post infection, d.p.i.). Shortly after infection (9 d.p.i.), cells could be divided into roughly two populations: effector T_FH_ (Bcl-6^HI^Cxcr5^HI^) and effector non-T_FH_ (Bcl-6^MID^Cxcr5^MID^). At this time point, the majority of the non-T_FH_ effector population expressed low levels of IL-7R. However, at later time points (30 and 60 d.p.i.), a substantial percentage of this population displayed increased expression of IL-7R (Fig. 7a). In comparison, T_FH_ cells expressed relatively low levels of IL-7R, especially at late time points post infection (Supplementary Fig. 4). Strikingly, the same cells that displayed increased expression of IL-7R also expressed the highest levels of IL-6Rα (Fig. 7b). Importantly, the IL-6Rα^+^IL-7R^+^ DP population increased with time post infection, whereas effector T_FH_ and non-T_FH_ (IL-7R^LO^) populations decreased (Fig. 7c). Collectively, these data indicate that a population of long-lived (60 d.p.i.), antigen-specific IL6Rα^+^IL7R^+^ DP cells arises during the post-influenza-infection period. Furthermore, the dual expression of IL-6Rα and IL-7R, in addition to our *in vitro* data demonstrating the differential regulation of T_FH_ and T_CM_ gene programs by IL-6 and IL-7, suggest that the activation of these cytokine-signalling pathways will likely influence the functional capabilities of the IL-6Rα^+^IL-7R^+^ cells *in vivo*.

## Discussion

Following infection, the contraction of the effector response and the subsequent emergence of the memory cell population are vital to long-lasting immunity. However, the potential for memory populations to arise from the numerous CD4^+^ T-cell subsets, as well as the degree of plasticity that exists between these subsets, has complicated the identification of specific environmental signals and precise transcriptional networks that direct the effector-to-memory transition. For example, T_FH_ and T_CM_ cells appear to share developmental pathways, including requirements for the transcriptional repressor Bcl-6 and low levels of IL-2 signalling^14^,^15^,^16^,^19^,^20^. Interestingly, our data suggest that the T_FH_ and T_CM_ gene programs can co-initiate from a population of effector T_H_1 cells upon increased Bcl-6 expression in response to IL-2 withdrawal, resulting in a ‘T_FH_/T_CM_-like' population. In agreement with these data, there is emerging evidence for a set of genes which are linked to both T_FH_ and memory cell development, including *Bcl6*, *Cxcr5*, *Il7r* and *Tcf7* (refs 9, 19, 34, 35, 36, 37, 38). In addition, these data are physiologically intuitive as IL-2 signalling decreases during the late stages of an immune response, coincident with the formation of memory cell populations. Indeed, both T_FH_ and T_CM_ cells are required post contraction to mediate long-lasting humoral- and cell-mediated immunity, respectively. Therefore, it is likely that, following the initial co-expression of the T_FH_ and T_CM_ gene programs, there are distinct environmental and transcriptional regulatory mechanisms that promote T_FH_ versus T_CM_-cell-dependent immune responses.

In support of this divergence, we observed that T_H_1 cells downregulate IL-2Rα while upregulating both IL-6Rα and IL-7R in response to a reduction in IL-2 signalling. Thus, these cells appear to reprogramme their ability to respond to both a T_CM_-essential (IL-7) and a T_FH_-associated (IL-6) cytokine. Our data further demonstrate that IL-6 treatment of T_FH_/T_CM_-like cells results in the further augmentation of the T_FH_ profile. However, IL-7 exposure results in an inhibition of T_FH_ genes, including Bcl-6. These findings highlight a previously unappreciated role for IL-7 in the repression of the T_FH_ gene program in post-effector T_H_1 cells. Importantly, they also suggest that Bcl-6 may only be required for the initiation of the T_CM_ gene program, and that further developmental steps require a unique and undefined set of transcriptional regulators. Indeed, there are many reports in the literature describing a reduction in Bcl-6 expression in T_CM_ cells at late time points post infection^7^,^13^,^19^,^30^,^57^. Furthermore, these findings are consistent with defined roles for Bcl-6 in the regulation of metabolism, cell cycle progression and apoptosis^39^,^46^,^47^,^51^,^52^,^58^,^59^. Thus, IL-7-dependent repression of Bcl-6 may ultimately be necessary to promote the unique sets of regulatory activities required for long-term memory cell survival.

Interestingly, while IL-6 treatment augmented T_FH_ gene expression, it did not affect the expression of T_CM_ genes in the T_FH_/T_CM_-like post-effector population. Thus, these cells express hallmark genes of both T_FH_ and T_CM_-cell types and resemble T_FH_-memory cells^50^. Whether there is a factor akin to IL-7 responsible for repressing the T_CM_-profile and promoting the T_FH_ cell fate is a question that remains to be answered. In support of the model proposed here and perhaps shedding light on this question, recent reports have highlighted the antagonistic nature of T_FH_-associated ICOS signalling and the T_CM_ transcription factor, Klf2. In these studies, ICOS signalling-dependent repression of Klf2 was required for commitment to the T_FH_-cell fate^60^,^61^. Thus, in addition to IL-6 signalling, interactions between T_FH_/T_CM_-like cells and B cells, along with the corresponding ICOS stimulation, are likely required to antagonize the T_CM_ gene program and specifically promote T_FH_-dependent immune responses.

Our data demonstrate that the IL-7-dependent repression of Bcl-6 is not a result of increased Blimp-1 expression. This is surprising, as these transcriptional repressors often appear in opposition during developmental steps in the immune system. Rather, our current data support a mechanism of transcriptional repression whereby activated STAT5 functions downstream of IL-7 signalling to repress Bcl-6 expression. This finding is similar to the inverse correlation between IL-2-dependent STAT5 activation and Bcl-6 expression that we reported in a prior study^12^. Interestingly, STAT5 has been shown to interact with the histone methyltransferase, Ezh2, downstream of IL-7 signalling^56^. As such, future experiments that determine whether there are cytokine-dependent differences to the composition of the STAT5 complex that binds to the *Bcl6* locus could provide insight into how IL-7 and IL-2 harbour non-redundant roles in the regulation of immune cell development and function, despite signalling through a common downstream transcriptional regulator, STAT5 (refs 54, 55).

Collectively, this study supports a model in which effector T_H_1 cells co-initiate T_FH_ and T_CM_ gene programs when IL-2 signals begin to wane. Our finding that antigen-specific effector CD4^+^ T cells upregulate the dual expression of IL-6Rα and IL-7R at late time points post influenza infection suggests that exposure to either cytokine could be a key determinant in further differentiation events and long-term cellular function. Although these findings may be indicative of divergence to either a T_FH_- or T_CM_-specific gene program, they do not preclude the possibility that plasticity exists between these cell states to influence trafficking between the T-cell and B-cell zones of secondary lymphoid tissues to provide a more comprehensive immune response. It is also possible that the simultaneous expression of IL-6Rα and IL-7R allow for precise modulation of Bcl-6 expression. A rheostatic model such as this may allow for modest Bcl-6 expression, sufficient to repress Blimp-1 expression and initiate the T_CM_ transcriptional program, while also repressing the enhanced levels of Bcl-6 required for T_FH_ development via IL-7 signalling. Future studies that address these possibilities by examining the functional properties of the IL-6Rα^+^IL-7R^+^ CD4^+^ cells described here will be critical to enhance our understanding of the relationship between the T_FH_ and T_CM_ cell types.

## Methods

### Primary cells and cell culture

Primary naive CD4^+^ T cells were isolated from the spleen and lymph nodes of sex- and age-matched (5–8-week old) wild-type C57BL/6 mice using the MagCellect kit (R&D, MAGM205), consistently providing a 90–95% pure population. Following isolation, cells were cultured on plate-bound αCD3/αCD28 in T_H_1 polarizing conditions (α-IL-4 (5 μg ml^−1^) and IL-12 (5 ng ml^−1^)). After 3 days, cells were removed from αCD3/αCD28 stimulation, split and cultured for an additional 2 days in either high (250 U ml^−1^) or low IL-2 (10 U ml^−1^) conditions to generate effector T_H_1 or T_FH_-like cells, respectively^12^. Low IL-2-treated cells were then treated with IL-6 (10 ng ml^−1^) or IL-7 (10 ng ml^−1^) unless otherwise indicated. Following a 24-h incubation, cells were collected for analysis. Primary T-cell transfections were performed with the 4D Lonza nucleofection system (program DN-100, solution P3). The Institutional Animal Care and Use Committees of Virginia Tech and the University of Alabama at Birmingham approved all the experimentation involving the use of mice. All the methods were performed in accordance with the approved guidelines.

Murine EL4 T cells (TIB-39, ATCC) were cultured in RPMI supplemented with 10% FBS and 1% Pen-strep. EL4 transfections were performed using the Lonza 4D nucleofection system (program CM-120, solution SF). Immunoblot analysis was used to assess the expression levels of all transfected proteins.

### RNA purification and quantitative PCR with reverse transcription

Cells were collected on day 5, 6 or 7 (dependent on experimental design) and RNA was purified using the Machery Nagel RNA purification kit. Complementary DNA was prepared using the First Strand Superscript II Synthesis System (Invitrogen). Quantitative PCR with reverse transcription (qRT–PCR) reactions were performed with the cDNA and gene-specific primers (Supplementary Table 1) and SYBR green master mix (Bio-Rad). For the experiments in Figs 1 and 5a, PrimePCR custom plates (Bio-Rad) were used. All the samples were normalized to the *Rps18* control with graphs representing data normalized to the indicated comparison condition.

### siRNA experiments

For the siRNA experiments, both control and *Prdm1*-specific siRNAs were obtained from Dharmacon (D-001210-01-20, D-043069; sequences in Supplementary Table 2). Primary T_H_1 cells were transfected with the indicated siRNAs on day 5. Following nucleofection with siRNA, primary cells were allowed to recover in high IL-2 conditions for 24 h before gene expression analysis. Efficiency of knockdown was determined by qRT–PCR and immunoblot analysis for *Prdm1* transcript and Blimp-1 protein expression, respectively.

### Immunoblot analysis

An equal number of cells were collected and subjected to immunoblot analysis to determine protein expression levels of Bcl-6 (BD Biosciences, 561520, dilution 1:500), Blimp-1 (Genscript, A01647, dilution 1:500), STAT5 (Santa Cruz, sc-835, dilution 1:5,000), phospho-STAT5 (pY694, BD Biosciences, 611964, dilution 1:5,000) and V5-tagged proteins (Invitrogen, R960-25, dilution 1:5,000). In brief, separation of lysates by SDS–polyacrylamide gel electrophoresis was followed by immunoblot analysis. GAPDH (Santa Cruz, sc-25778, dilution 1:2,500) or β-actin (Genscript, A00730, dilution 1:10,000) expression was monitored to ensure equal protein loading. Additional information for antibodies can be found in Supplementary Table 3 and uncropped versions of all immunoblots are provided in Supplementary Fig. 5.

### ChIP assay

The ChIP assay was performed as published^12^. In brief, chromatin was harvested from T_FH_/T_CM_-like cells treated with and without IL-7 as indicated. Chromatin was incubated with antibodies to either STAT5 (Santa Cruz, sc-835x, 5 μg per IP) or IgG (Abcam, ab6709, 5 μg per IP) control and the precipitated DNA was analysed by qPCR with gene-specific primers (Supplementary Table 1). Samples were normalized to a standardized total input DNA control followed by subtraction of the IgG antibody as a control for the nonspecific background. The final value represents the percent enrichment of STAT5-specific signal.

### Promoter–reporter analysis

S*ell* (−1,755 to +79 bp), *Ccr7* (−1,470 to +113 bp) and *Tbx21* (−1,893 to +194 bp) promoter–reporter vectors were prepared by cloning regulatory regions of each gene into the pGL3-basic luciferase reporter construct (Promega). EL4 cells were co-transfected with the promoter–reporter constructs in combination with the indicated expression vectors as well as a TK-*renilla* control plasmid (Promega) to normalize for transfection efficiency. Transfections were harvested after 16–24 h and samples were analysed with the Dual-Luciferase Reporter system (Promega).

### Flow cytometry

Fluorochrome-labelled α-IL-6Rα (P22272, dilution 1:10), α-IL-7R (A7R34, dilution 1:10), α-CD62L (95218, dilution 1:20), α-Cxcr5 (614641, dilution 1:10) and respective isotype control antibodies were purchased from R&D. α-Ccr7 (4B12, dilution 1:50), α-IL-2Rα (PC61.5, dilution 1:50), α-IL-7R (SB/199, dilution 1:10) and α-CD62L (MEL-14, dilution 1:100) were purchased from eBioscience. Non-viable cells were excluded using e520 or e450 viability dye (eBioscience), Sytox Green or Blue (Life Technologies) or propidium iodide (BD Biosciences). For the staining procedure, cells were collected on day 6 (3 days post transition to low IL-2 conditions) and subjected to labelling and sorting as indicated. Briefly, cells were pelleted, washed in 500 μl of 1 × FACS buffer (2% FBS, 1% BSA, 0.1% NaNH_3_), and then stained with indicated fluorochrome-conjugated antibody. Following staining with fluorochrome-conjugated antibodies and viability dye, cells were washed three times in 1 × FACS buffer and then resuspended for analysis on either an Accuri C6 flow cytometer or Sony SH800. All the obtained data were analysed using FlowJo Software.

### Cell sorting and droplet digital PCR

Day 6 IL-6Rα^+^/IL-7R^+^ DP cells were sorted (∼500,000 cells) using a Sony SH800 using a 100 μm chip under purity-sort mode and cDNA was prepared as described above (see qRT–PCR). Droplet Digital PCR reactions were performed using gene-specific primers diluted in 2 × master mix (Qx200 ddPCR EvaGreen Supermix, Bio-Rad). Droplets were generated using a Bio-Rad Automated Droplet Generator and end-point PCR was performed. Droplets were then read using a Qx200 Digital PCR Reader using QuantaSoft Software (Bio-Rad). All the primers were diluted to optimize signal-to-noise and thresholds were set using non-template control wells. Absolute counts were normalized to *Rps18* and then fold change was determined.

### Influenza virus infections and *in vivo* analysis

Influenza virus infections were performed intranasally with 6,500 VFU of A/PR8/34 (PR8) in 100 μl of PBS. Cell suspensions from mLNs were prepared by passing tissues through nylon mesh. Cells from mLNs were resuspended in 150 mM NH_4_Cl, 10 mM KHCO_3_ and 0.1 mM EDTA for 5 min to lyse red cells. Cell suspensions were then filtered through a 70 μm nylon cell strainer (BD Biosciences), washed and resuspended in PBS with 5% donor calf serum and 10 μg ml^−1^ FcBlock (2.4G2 -BioXCell) for 10 min on ice before staining with fluorochrome-conjugated antibodies or tetramer reagents. Fluorochrome-labelled α-PD-1 (J43, dilution 1:100), α-IL-7R (A7R34, dilution 1:100) and α-IL-6Rα (D7725A7, dilution 1:100) were from eBioscience. Fluorochrome-labelled α-Bcl-6 (K112.91, dilution 1:50), α-Cxcr5 (2G-8, dilution 1:50) and α-CD4 (RM4-5, dilution 1:200) were from BD Biosciences. The IA^b^NP_311-325_ MHC class II tetramer was obtained from the NIH Tetramer Core Facility and used at a 1:100 dilution. Intracellular staining for Bcl-6 was performed using the mouse regulatory T-cell staining kit (eBioscience) following the manufacturer's instructions. Flow cytometry was performed using a FACSCanto II (BD Biosciences) and analysed in FlowJo.

### Statistics

All the data represent at least three independent experiments with the number of biological replicates indicated in the figure legend. Error bars represent the s.e.m. or s.d. as indicated. For statistical analysis, an unpaired *t* test was performed using GraphPad Prism online software. *P* values <0.05 were considered statistically significant.

## Additional information

**How to cite this article:** McDonald, P. W. *et al*. IL-7 signalling represses Bcl-6 and the T_FH_ gene program. *Nat. Commun.* 7:10285 doi: 10.1038/ncomms10285 (2016).

## Supplementary Material

Supplementary Information Supplementary Figures 1-5 and Supplementary Tables 1-3
