# Supplementary material for: IL-7 signalling represses Bcl-6 and the TFH gene program
Source: Nat Commun. 2016 Jan 8;7:10285. doi: 10.1038/ncomms10285 (PMC4729877; doi:10.1038/ncomms10285)
Supplement: Supplementary Information — Supplementary Figures 1-5 and Supplementary Tables 1-3 [file ncomms10285-s1.pdf]

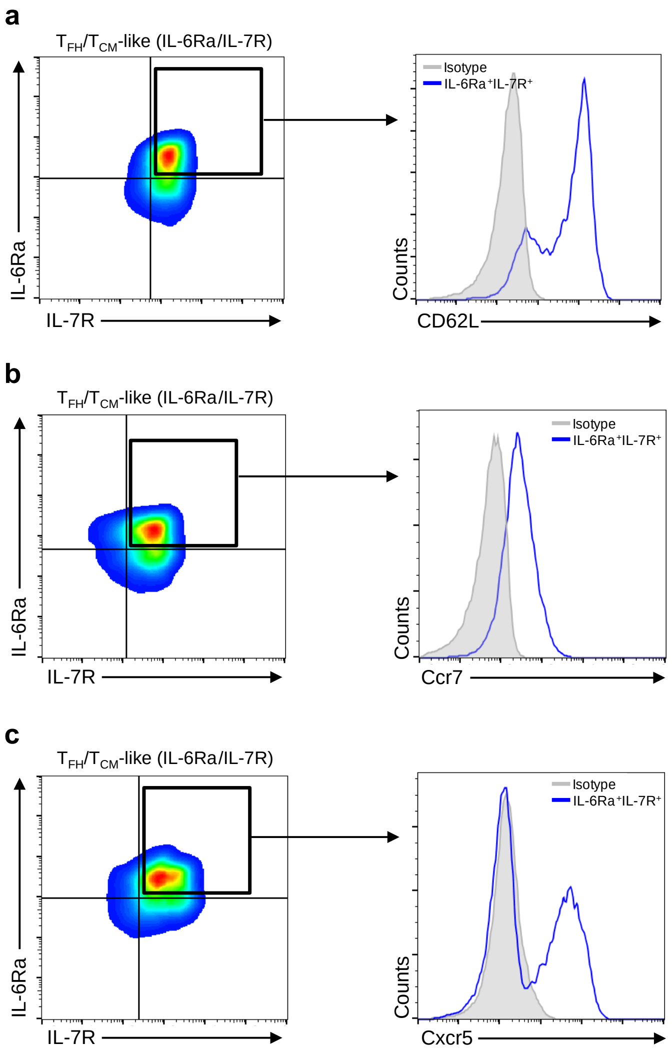

Supplementary Figure 1. IL-6Rα^+IL-7R$^+$ cells express CD62L, Ccr7, and Cxcr5.

Primary CD4$^+$ T cells were cultured in T$_H$1 conditions and exposed to a low concentration of IL-2 (10 U/ml). On day 5, cell surface expression of IL-6Rα and IL-7R was measured by flow cytometric analysis. Subsequently, IL-6Rα^+IL-7R$^+$ cells were gated and assessed for expression of (a) CD62L, (b) Ccr7, or (c) Cxcr5. Shown are representative histogram plots of two independent experiments performed.

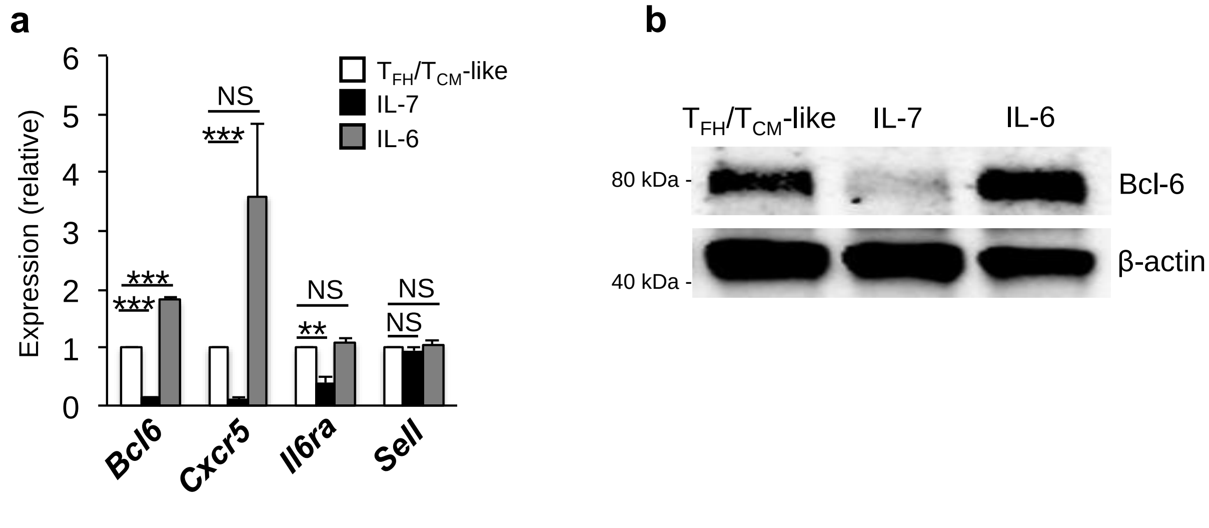

Supplementary Figure 2. IL-7 signaling represses Bcl-6 and additional T_{FH} genes.

Primary $CD4^+$ T cells were cultured in T_H1 conditions and exposed to either high or low IL-2 conditions to generate effector T_H1 or T_{FH}/T_{CM}-like (IL-6Rα^+IL-7R$^+$) cells, respectively. T_{FH}/T_{CM}-like cells were then exposed to IL-7. Following a 24-hour incubation, expression of the indicated genes was analyzed by (**a**) qRT-PCR or (**b**) immunoblot. For **a**, the sample values were compared relative to the T_{FH}/T_{CM}-like sample for each independent experiment and expressed as fold expression (mean of $n = 3 \pm$ s.e.m.). For **b**, Bcl-6 protein expression was measured with β-actin serving as a loading control. Shown is a representative blot of three independent experiments performed. $^{**}P < 0.01$, $^{***}P < 0.001$ (unpaired Student's *t*-test).

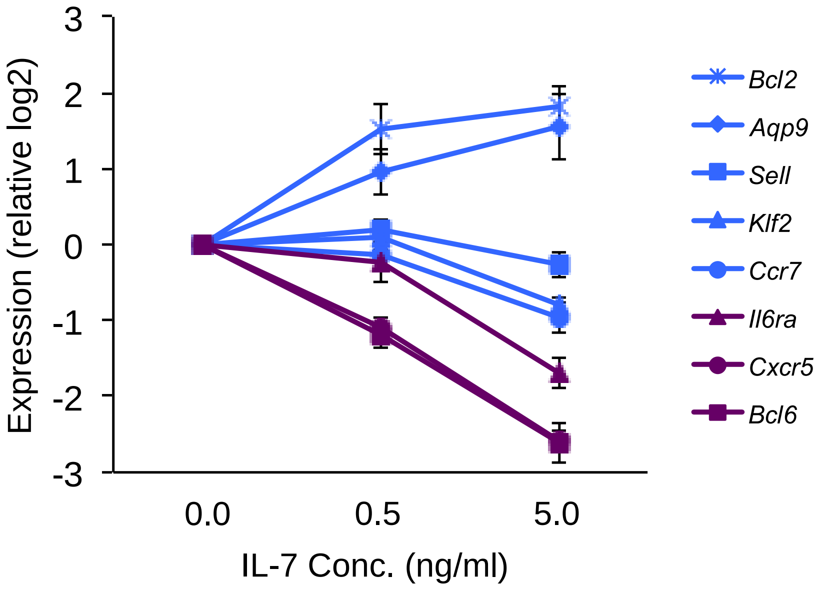

Supplementary Figure 3. T_{FH} genes are preferentially repressed at low concentrations of IL-7. T_{FH}/T_{CM}-like cells were generated as described in Supplementary Figure 2 and exposed to the indicated concentration of IL-7. Following a 24-hour incubation, expression of the indicated genes was assessed via qRT-PCR. The sample values were compared relative to the untreated T_{FH}/T_{CM}-like cell for each independent experiment and expressed as the relative log2 change (>2 fold reduction, purple; <2 fold reduction, blue; mean of n = 3 ± s.e.m.).

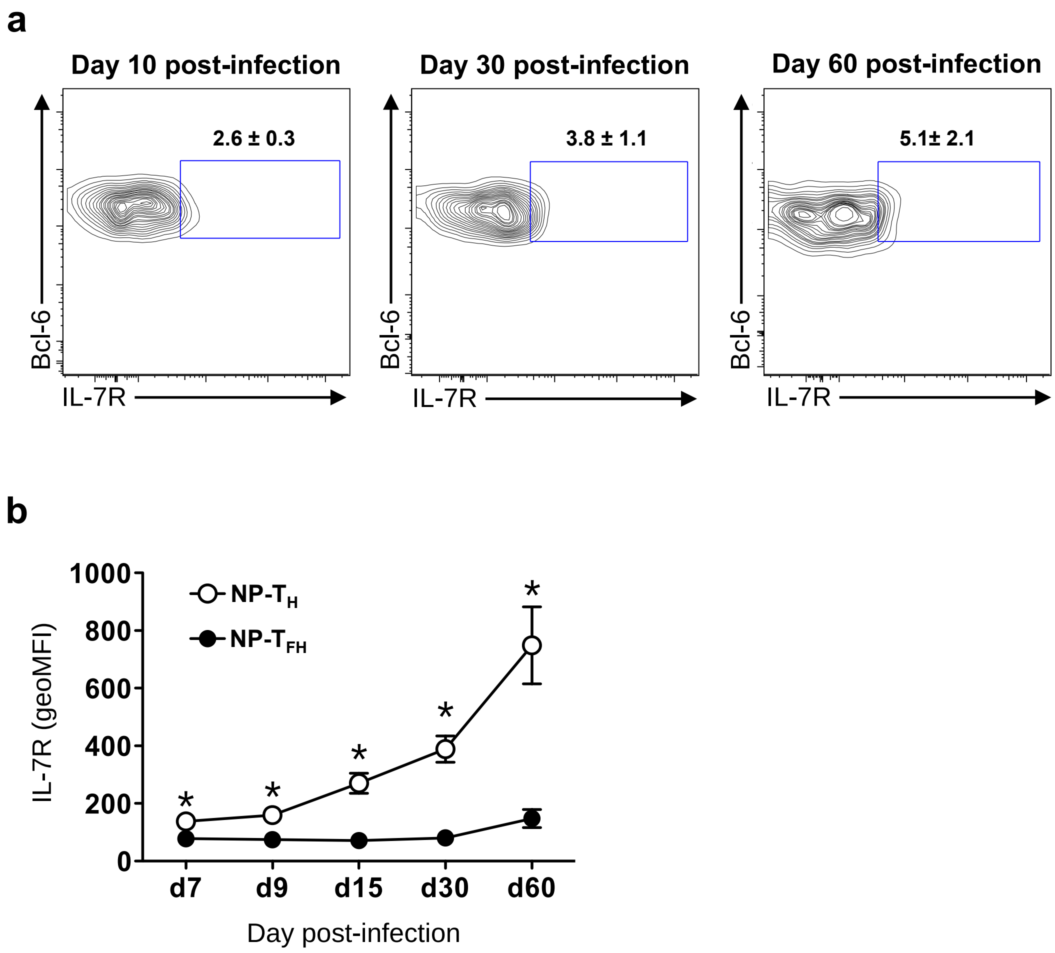

Supplementary Figure 4. Influenza-specific T_{FH} cells express low levels of IL-7R.

Mice were infected with influenza (PR8) and $CD4^{+}CD19^{-}Foxp3^{-}$ cells were analyzed by flow cytometric analysis at the indicated day post-infection. (**a**) Nucleoprotein (NP)-specific cells were isolated and T_{FH} ($Bcl\text{-}6^{HI}Cxcr5^{HI}$) cells were assessed for IL-7R expression at the indicated day post-infection. (**b**) IL-7R (geometric mean fluorescence intensity) expression was measured for NP-specific T_{FH} (filled circles) and T_{H} (open circles) populations post-influenza infection (mean ± s.d. of 4-5 mice per group, *$P < 0.001$, two-tailed Student's t-test).

Figure 1c

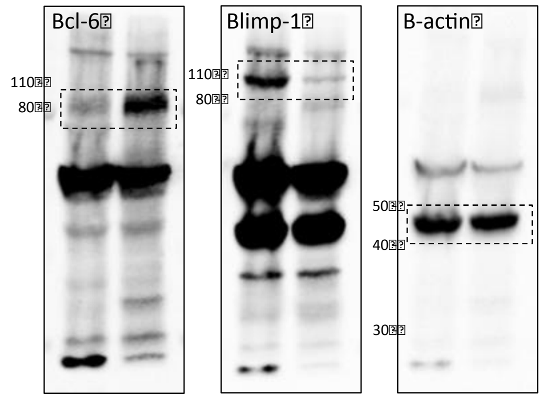

Figure 2b

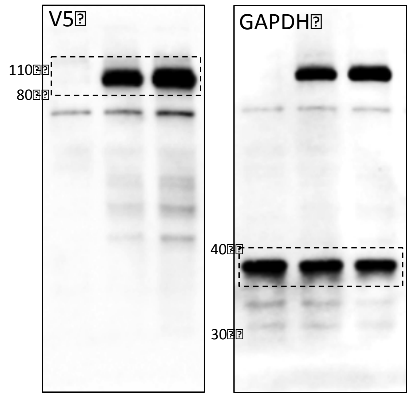

Figure 2d

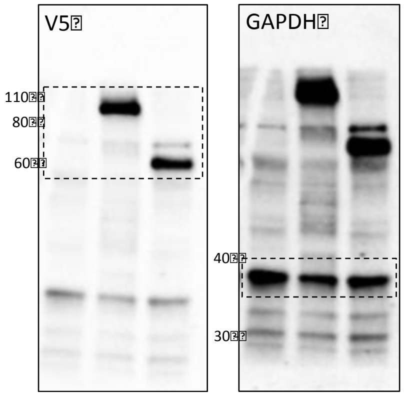

Figure 2g

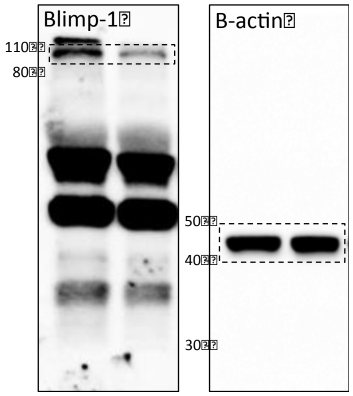

Supplementary Figure 5. Original and uncropped scans of immunoblots (Part I).

Figure 5b

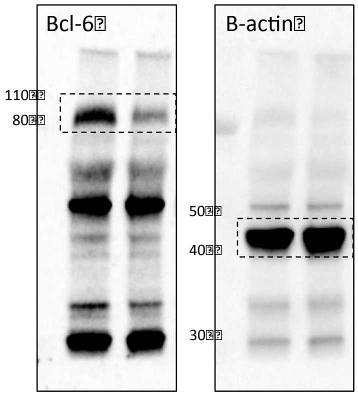

Supplementary Figure 2b

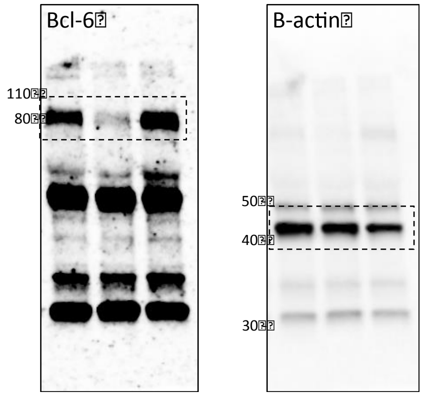

Figure 6b

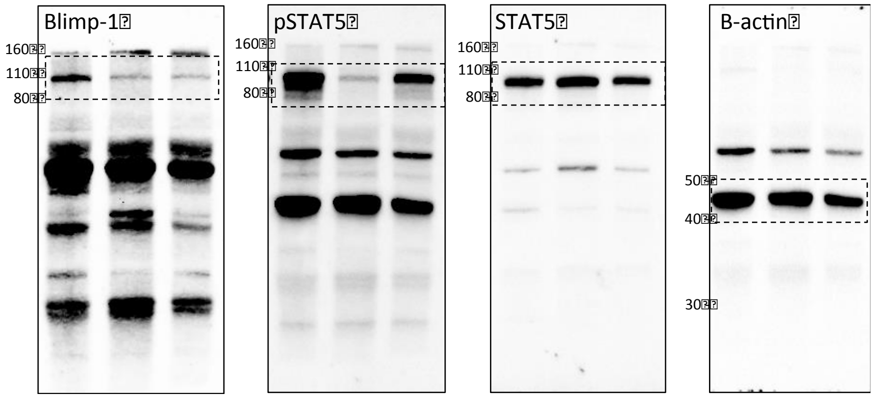

Figure 6c

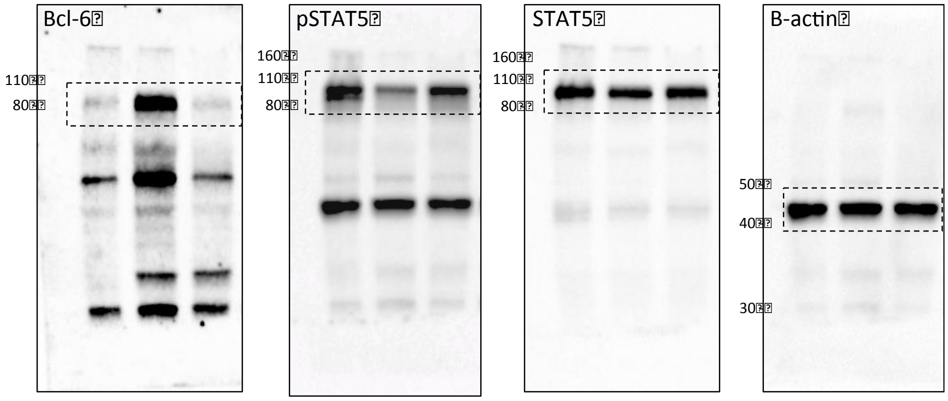

Supplementary Figure 5. Original and uncropped scans of immunoblots (Part II).

Supplementary Table 1. Primer sequences

qRT-PCR primer sequences

Gene	Species	Sequence (5' to 3')
Bcl6	mouse	fwd: CCAACCTGAAGACCCACACTC<br>rev: GCGCAGATGGCTCTTCAGAGTC
Cxcr5	mouse	fwd: GTACCTAGCCATCGTCCATGC<br>rev: GTGCACTGTGGTAAGGAGTCG
Cxcr5 (ddPCR only)	mouse	proprietary, Bio-Rad qMmuCED0039913
IL6ra	mouse	fwd: CCACATAGTGTCACTGTGCG<br>rev: GGTATCGAAGCTGGAACTGC
Prdm1	mouse	fwd: CTTGTGTGGTATTGTCGGGAC<br>rev: CACGCTGTACTCTCTCTTGG
Sell	mouse	fwd: GAGGGTACTTACTGGGGCTC<br>rev: GCATTTTCCCAGTTCATGGG
Ccr7	mouse	fwd: GGCTAGCTGGAGAGAGACAAGAACC<br>rev: GACAAGGAGAGCCACCACCAGC
Klf2	mouse	fwd: GACCTACACCAAGAGCTCGC<br>rev: CTGTGTGCTTTCGGTAGTGG
Aqp9	mouse	fwd: CGAGCCAAGAAGAACCTCGTGCAG<br>rev: CTGAGGACTGCTTGAGCAATAGAGC
Bcl2	mouse	fwd: GTCAACAGGGAGATGTCACC<br>rev: GGCCATATAGTTCCACAAAGGC

ChIP primer sequences

Gene	Species	Sequence
Bcl6 set "A"	mouse	fwd: GTACTCCAACAACAGCACAGC<br>rev: GTGGCTCGTTAAATCACAGAGG
Bcl6 set "B"	mouse	fwd: GCGGAGCAATGGTAAAGCCC<br>rev: CTGGTGTCCGGCCTTTCCTAG

Supplementary Table 2. siRNA sequences

siRNA sequences targeting murine genes (purchased from Dharmacon)

siRNA Name (siGENOME Mouse Prdm1)	Target Sequence
D-043069-09	GGAGAGACCCACCUACAUA
D-043069-10	GCAAUACAGUAGUGAGAAA
D-043069-11	GGAAGGACAUCUACCGUUC
D-043069-21	GUACAUACAUAGUGAACGA
siGENOME non-targeting siRNA #1 (D-001210-01-20)	UAGCGACUAAACACAUCAA

Supplementary Table 3. Antibodies

Primary antibodies

Antibody	Dilution/Concentration	Species	Clone	Catalog#	Source
b-actin:HRP	1:10,000 (WB)	mouse	2D1D10	A00730	Genscript
Bcl-6	1:500 (WB)	mouse	K112-91	561520	BD Biosciences
Blimp-1	1:500 (WB)	rabbit		A01647	Genscript
STAT5 (C-17)	1:5000 (WB) / 5ug/IP	rabbit		sc-835X	Santa Cruz
pSTAT5 (pY694)	1:5000 (WB)	mouse	47/Stat5(pY694)	611964	BD Biosciences
GAPDH (FL-335)	1:2500 (WB)	rabbit		sc-25778	Santa Cruz
V5	1:5000 (WB)	mouse		R960-25	Invitrogen
